# Supplementary material for: A microsporidian impairs Plasmodium falciparum transmission in Anopheles arabiensis mosquitoes
Source: Nat Commun. 2020 May 4;11:2187. doi: 10.1038/s41467-020-16121-y (PMC7198529; doi:10.1038/s41467-020-16121-y)
Supplement: Supplementary file 1 — Supplementary Information [file 41467_2020_16121_MOESM1_ESM.docx]

Supplementary Information for “A Microsporidian impairs *Plasmodium falciparum* transmission in *Anopheles arabiensis* mosquitoes”

Herren et al.

Supplementary Figures


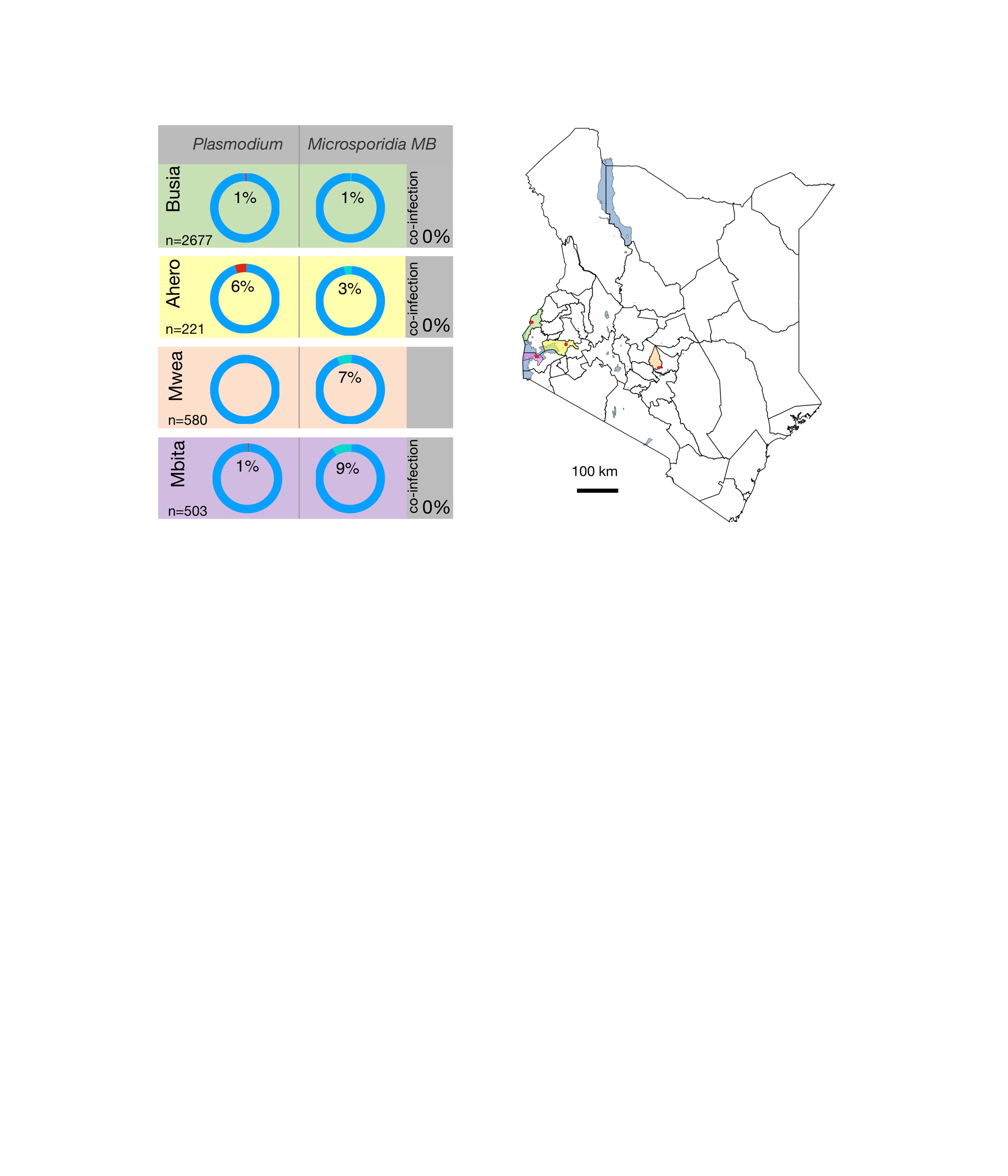


Supplementary Figure 1: *Microsporidia MB* is found in geographically dispersed *An. arabiensis* populations. *Microsporidia MB* is found at a prevalence range of <1-9% in *An. arabiensis* populations that also have *Plasmodium* sporozoite infections at a prevalence range of 1-6%. *Microsporidia MB* and *Plasmodium* sporozoite co-infections were not observed. The color-coded locations of sampling sites are indicated on the map of Kenya. Source data are provided as a Source Data file. Map generated using QGIS version 3.6.2 without any changes (QGIS Development Team, 2016. QGIS Geographic Information System. Open Source Geospatial Foundation Project. <http://www.qgis.org/>). Map is licensed under Creative Commons Attribution-ShareAlike 3.0 licence (CC BY-SA) https://creativecommons.org/licenses/by-sa/3.0/.

Supplementary Figure 2: The RNA polymerase B' subunit gene (*rpoB*) based phylogeny reveals that *Microsporidia MB* are in clade IV of the Microsporidia. *Microsporidia MB* *rpoB* gene sequence is available in Source Data file.


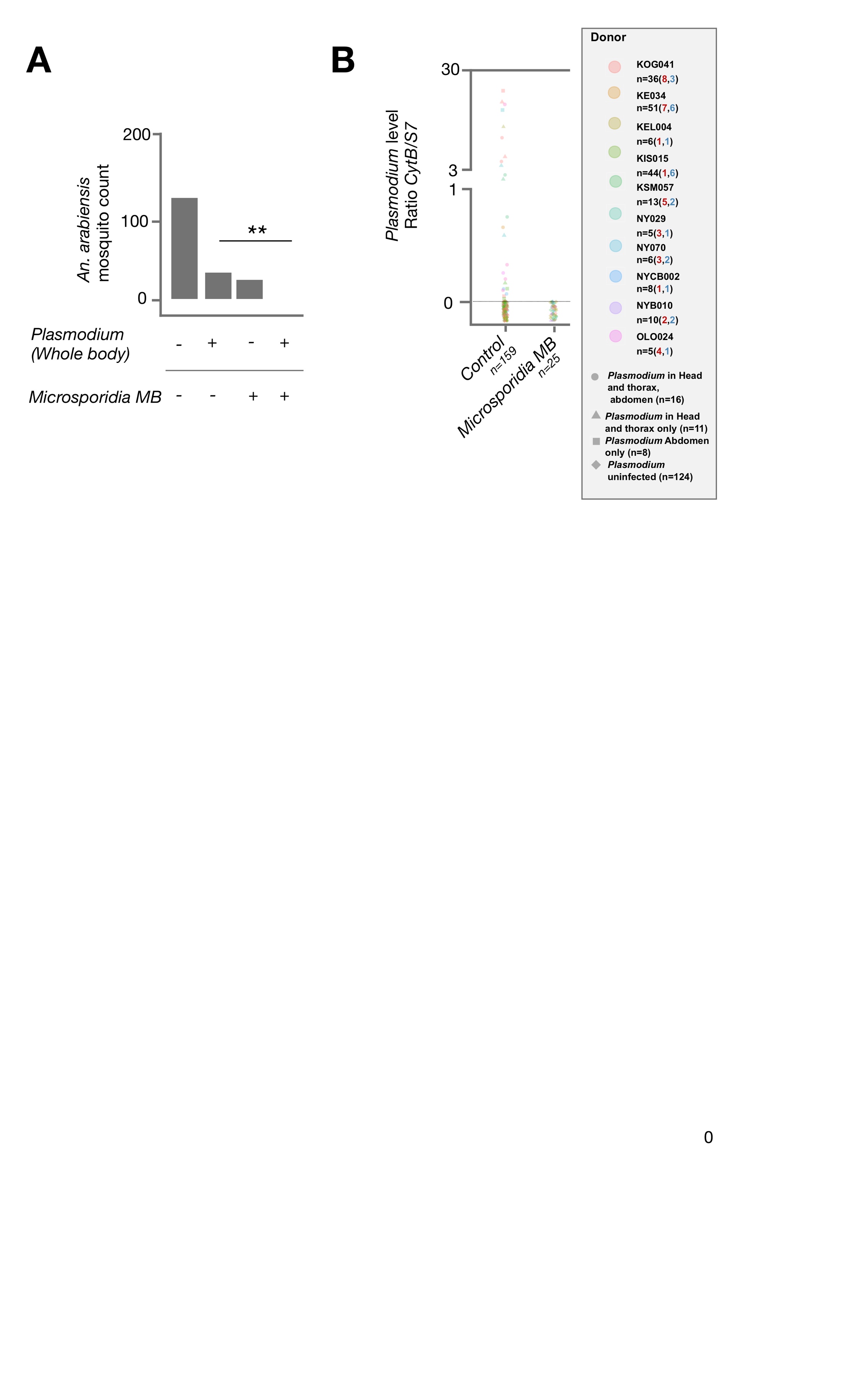


Supplementary Figure 3: *Microsporidia MB* impairs *Plasmodium falciparum* transmission in *An. arabiensis* after direct membrane feeding assay challenge. The *Plasmodium* infection rate in *Microsporidia MB* positive and *Microsporidia MB* negative mosquitoes was determined by qPCR*.* (A) The whole mosquito *Plasmodium* infection rate in *Microsporidia MB* positive and *Microsporidia MB* negative mosquitoes. There was a significant absence of co-infected mosquitoes (two tailed fisher exact test, P=0.005 n=184). (B) *Plasmodium* density quantified by qPCR in *An. arabiensis.* For *An. arabiensis* mosquitos infected in both head and thorax and abdomen, average *Plasmodium* density across both compartments is shown*.* If *Plasmodium* was detected in either head and thorax or abdomens or both compartments, the *An. arabiensis* was considered to have a *Plasmodium* infection. Source data are provided as a Source Data file.

Supplementary Tables

| **Mosquito ID** | **Donor ID** | **Microscopy Oocyst count** | **qPCR *Plasmodium* levels Ratio CytB/S7** |
| --- | --- | --- | --- |
| AAKE002.29 | KE002 | 32 | 51.8660 |
| AAKE002.27 | KE002 | 31 | 8.8097 |
| AAKE002.17 | KE002 | 27 | 2.6563 |
| AAKE002.20 | KE002 | 26 | 3.1649 |
| AAKE002.22 | KE002 | 17 | 12.3944 |
| AAKE002.26 | KE002 | 16 | 0.1170 |
| AAKE002.32 | KE002 | 14 | 5.5038 |
| AAKE002.13 | KE002 | 14 | 6.2848 |
| AAKE002.25 | KE002 | 13 | 16.2045 |
| AAKE002.12 | KE002 | 10 | 0.3178 |
| AAKE002.18 | KE002 | 4 | 0.2263 |
| AAKE002.16 | KE002 | 4 | 0.2646 |
| AAKE002.30 | KE002 | 3 | 0.0000 |
| AAKE002.11 | KE002 | 3 | 19.1503 |
| AAKE002.23 | KE002 | 2 | 0.0192 |
| AAAL106.19 | AL106 | 1 | 0.0000 |
| AAAL106.7 | AL106 | 1 | 0.0291 |
| AAKE002.24 | KE002 | 1 | 0.0381 |
| AAAL106.11 | AL106 | 1 | 0.0723 |
| AAAL106.18 | AL106 | 1 | 0.1401 |
| AAKE002.21 | KE002 | 1 | 0.3971 |
| AAAL106.1 | AL106 | 0 | 0.0000 |
| AAAL106.2 | AL106 | 0 | 0.0000 |
| AAAL106.3 | AL106 | 0 | 0.0000 |
| AAAL106.4 | AL106 | 0 | 0.0000 |
| AAAL106.5 | AL106 | 0 | 0.0000 |
| AAAL106.6 | AL106 | 0 | 0.0000 |
| AAAL106.8 | AL106 | 0 | 0.0000 |
| AAAL106.9 | AL106 | 0 | 0.0000 |
| AAAL106.10 | AL106 | 0 | 0.0000 |
| AAAL106.12 | AL106 | 0 | 0.0000 |
| AAAL106.13 | AL106 | 0 | 0.0000 |
| AAAL106.14 | AL106 | 0 | 0.0000 |
| AAAL106.15 | AL106 | 0 | 0.0000 |
| AAAL106.16 | AL106 | 0 | 0.0000 |
| AAAL106.17 | AL106 | 0 | 0.0000 |
| AAAL106.20 | AL106 | 0 | 0.0000 |
| AAAL106.22 | AL106 | 0 | 0.0000 |
| AAAL106.23 | AL106 | 0 | 0.0000 |
| AAAL106.24 | AL106 | 0 | 0.0000 |
| AAAL106.26 | AL106 | 0 | 0.0000 |
| AAAL106.27 | AL106 | 0 | 0.0000 |
| AAAL106.28 | AL106 | 0 | 0.0000 |
| AAGA034.1 | GA034 | 0 | 0.0000 |
| AAGA034.2 | GA034 | 0 | 0.0000 |
| AAGA034.3 | GA034 | 0 | 0.0000 |
| AAGA034.4 | GA034 | 0 | 0.0000 |
| AAGA034.6 | GA034 | 0 | 0.0000 |
| AAGA034.7 | GA034 | 0 | 0.0000 |
| AAGA034.8 | GA034 | 0 | 0.0000 |
| AAGA034.9 | GA034 | 0 | 0.0000 |
| AAGA034.10 | GA034 | 0 | 0.0000 |
| AAGA034.11 | GA034 | 0 | 0.0000 |
| AAGA034.12 | GA034 | 0 | 0.0000 |
| AAGA034.13 | GA034 | 0 | 0.0000 |
| AAKE002.1 | KE002 | 0 | 0.0000 |
| AAKE002.2 | KE002 | 0 | 0.0000 |
| AAKE002.3 | KE002 | 0 | 0.0000 |
| AAKE002.4 | KE002 | 0 | 0.0000 |
| AAKE002.5 | KE002 | 0 | 0.0000 |
| AAKE002.6 | KE002 | 0 | 0.0000 |
| AAKE002.7 | KE002 | 0 | 0.0000 |
| AAKE002.8 | KE002 | 0 | 0.0000 |
| AAKE002.9 | KE002 | 0 | 0.0000 |
| AAKE002.10 | KE002 | 0 | 0.0000 |
| AAKE002.31 | KE002 | 0 | 0.0000 |
| AAKE002.33 | KE002 | 0 | 0.0000 |
| AAKE002.34 | KE002 | 0 | 0.0000 |
| AAAL106.21 | AL106 | 0 | 0.0016 |
| AAAL106.25 | AL106 | 0 | 0.0026 |
| AAKE002.15 | KE002 | 0 | 0.0516 |
| AAKE002.28 | KE002 | 0 | 0.0588 |
| AAKE002.14 | KE002 | 0 | 0.4775 |
| AAGA034.5 | GA034 | 0 | 2.3810 |
| AAKE002.19 | KE002 | 0 | 14.3188 |

Supplementary Table 1: Comparison between microscopy and qPCR-based identification of *Plasmodium* oocysts.

Transcript count

| **Vectorbase ID** | **Annotation** | **Control midgut** | ***Microsporidia MB* midgut** |
| --- | --- | --- | --- |
| AARA001692-RA | \|protein_coding\|KB704673:1533830:1534351:1 | 4 | 1427 |
| AARA001672-RA | serine protease\|protein_coding\|KB704673:1206862:1207866:1\| | 0 | 863 |
| AARA017919-RA | \|protein_coding\|KB704485:2440621:2441697:-1\| | 0 | 684 |
| AARA010474-RA | \|protein_coding\|KB704784:4788208:4788865:-1\| | 0 | 668 |
| AARA016538-RA | trypsin\|protein_coding\|KB704562:10018691:10019618:-1\| | 0 | 606 |
| AARA011947-RA | \|protein_coding\|KB704585:216071:219571:1\| | 4 | 585 |
| AARA009400-RA | female reproductive tract protease GLEANR_896\|protein_coding\|KB704429:955380:956226:-1\| | 2 | 544 |
| AARA016534-RA | Tryp1: trypsin serine protease\|protein_coding\|KB704562:10011635:10012589:-1\| | 3 | 470 |
| AARA015679-RA | CEC2 protein\|protein_coding\|KB704784:2597926:2598493:1\| | 1 | 444 |
| AARA010954-RA | GAM1: Gambicin\|protein_coding\|KB704562:4778215:4779020:-1\| | 0 | 413 |
| AARA008584-RA | chymotrypsin\|protein_coding\|KB704237:5970022:5970611:1\| | 4 | 402 |
| AARA005220-RA | Spink6\|protein_coding\|KB704374:4924358:4925022:1\| | 0 | 389 |
| AARA001662-RA | \|protein_coding\|KB704673:1151138:1152222:1\| | 5 | 328 |
| AARA010362-RA | cecropin anti-microbial peptide\|protein_coding\|KB704784:2593765:2594023:-1\| | 0 | 299 |
| AARA001649-RA | \|protein_coding\|KB704673:748599:749200:-1\| | 3 | 279 |
| AARA014775-RA | peritrophin\|protein_coding\|KB704452:1249892:1250418:-1\| | 1 | 273 |
| AARA002670-RA | \|protein_coding\|KB705117:892980:893603:-1\| | 1 | 245 |
| AARA003942-RA | \|protein_coding\|KB705006:5236596:5236931:-1\| | 0 | 228 |
| AARA009129-RA | peritrophin\|protein_coding\|KB704452:1244597:1245158:-1\| | 1 | 210 |
| AARA008305-RA | \|protein_coding\|KB704237:552614:554459:1\| | 1 | 207 |
| AARA004201-RA | \|protein_coding\|KB704451:2368184:2368990:1\| | 0 | 191 |
| AARA008319-RA | \|protein_coding\|KB704237:810943:812029:1\| | 0 | 166 |
| AARA015903-RA | \|protein_coding\|KB704451:6659776:6660325:-1\| | 0 | 144 |
| AARA016215-RA | \|protein_coding\|KB704407:3555520:3556405:1\| | 1 | 136 |
| AARA015784-RA | \|protein_coding\|KB704348:4256032:4258087:-1\| | 0 | 126 |
| AARA018436-RA | \|protein_coding\|KB704385:4202700:4205032:1\| | 2 | 122 |
| AARA017081-RA | \|protein_coding\|KB704418:4431242:4433340:1\| | 0 | 119 |
| AARA008295-RA | chitinase\|protein_coding\|KB704237:383112:384913:-1\| | 0 | 111 |
| AARA017523-RA | carboxypeptidase A\|protein_coding\|KB704562:8559544:8561224:-1\| | 1 | 101 |
| AARA008583-RA | Chymotrypsin-1\|protein_coding\|KB704237:5958609:5959617:1\| | 1 | 95 |
|  |  |  |  |

Supplementary Table 2: List of genes with highest transcript count in *Microsporidia MB*-infected midguts. A full list of genes is provided as a Source Data file.

Transcript count

| **Vectorbase ID** | **Annotation** | **Control Ovary** | ***Microsporidia MB* Ovary** |
| --- | --- | --- | --- |
| AARA016504-RA | LYSC2: C type lysozyme\|protein_coding\|KB704125:12997130:12997706:-1\| | 0 | 145 |
| AARA010742-RA | Tsf1: Transferrin\|protein_coding\|KB704784:9541677:9544708:-1\| | 2 | 74 |
| AARA000121-RA | \|protein_coding\|KB704629:627908:628999:-1\| | 0 | 68 |
| AARA001017-RA | SG6: salivary gland protein 6\|protein_coding\|KB704474:1139482:1139829:1\| | 2 | 62 |
| AARA001523-RA | \|protein_coding\|KB704126:5724349:5724921:-1\| | 8 | 61 |
| AARA002817-RA | \|protein_coding\|KB705117:5969995:5970243:1\| | 0 | 57 |
| AARA008387-RA | salivary gland protein\|protein_coding\|KB704237:1947776:1948202:-1\| | 0 | 54 |
| AARA018276-RA | \|protein_coding\|KB704629:1332902:1334509:1\| | 0 | 52 |
| AARA016177-RA | salivary gland protein 2-like\|protein_coding\|KB704237:1942298:1942986:-1\| | 0 | 51 |
| AARA014717-RA | salivary gland protein\|protein_coding\|KB704237:1948586:1949299:1\| | 0 | 50 |
| AARA015677-RA | \|protein_coding\|KB704485:351430:351866:-1\| | 0 | 36 |
| AARA017351-RA | translation initiation factor 4E\|protein_coding\|KB704125:10819264:10823225:1\| | 5 | 32 |
| AARA015944-RB | myosin heavy chain\|protein_coding\|KB704125:14151741:14196446:1\| | 4 | 29 |
| AARA008372-RA | \|protein_coding\|KB704237:1784410:1784685:-1\| | 0 | 27 |
| AARA014082-RA | \|protein_coding\|KB704348:4511294:4513913:-1\| | 1 | 27 |
| AARA016239-RA | D7 short form salivary protein\|protein_coding\|KB704562:10085174:10085895:1\| | 0 | 25 |
| AARA016236-RA | D7 short form salivary protein\|protein_coding\|KB704562:10086838:10087593:1\| | 0 | 24 |
| AARA005793-RA | zinc finger protein\|protein_coding\|KB704895:8632779:8633012:1\| | 4 | 23 |
| AARA017267-RA | serine protease snake\|protein_coding\|KB704607:1737540:1738867:1\| | 0 | 22 |
| AARA000154-RA | \|protein_coding\|KB704629:1260841:1267015:-1\| | 2 | 22 |
| AARA007305-RA | \|protein_coding\|KB704125:1421292:1422270:-1\| | 2 | 22 |
| AARA015678-RA | \|protein_coding\|KB704485:349362:349737:-1\| | 0 | 21 |
| AARA016439-RA | long wavelength sensitive opsin\|protein_coding\|KB704673:689026:690578:-1\| | 0 | 21 |
| AARA005456-RA | 5' nucleotidase, ecto\|protein_coding\|KB704895:1659094:1661257:-1\| | 1 | 21 |
| AARA007307-RA | serine/threonine-protein phosphatase dullard homolog\|protein_coding\|KB704125:1425530:1435778:-1\| | 3 | 21 |
| AARA005602-RA | \|protein_coding\|KB704895:5042392:5043204:1\| | 3 | 20 |
| AARA004183-RA | small nuclear ribonucleoprotein polypeptide F\|protein_coding\|KB704451:2078616:2079165:-1\| | 2 | 19 |
| AARA005298-RA | \|protein_coding\|KB704696:207749:208932:1\| | 3 | 19 |
| AARA015837-RA | DLP: Daxx-like protein\|protein_coding\|KB704485:337567:342065:-1\| | 3 | 19 |

Supplementary Table 3: List of genes with highest transcript count in *Microsporidia MB*-infected ovaries. A full list of genes is provided as a Source Data file.

| **Species ID** | **Gene** | **Genbank Accession number** |
| --- | --- | --- |
| *Conidiobolus osmodes* | *18S* | AF368510 |
| *Encephalitozoon intestinalis* | *18S* | XR_002670134.1 |
| *Nosema ceranae* | *18S* | XR_002966746.1 |
| *Vittaforma corneae* | *18S* | U11046 |
| *Crispospora chironomi* | *18S* | ﻿GU130407 |
| *Anncaliia algerae* | *18S* | HM216911 |
| *Edhazardia aedis* | *18S* | AF027684 |
| *Parathelohania anophelis* | *18S* | AF027682 |
| *Hazardia milleri* | *18S* | AY090067 |
| *Conidiobolus osmodes* | *rpoB* | EF392431 |
| *Nosema ceranae* | *rpoB* | XM_024473662 |
| *Vittaforma corneae* | *rpoB* | XM_007605356 |
| *Encephalitozoon intestinalis* | *rpoB* | XM_003073661 |
|  |  |  |

Supplementary Table 4: Accession numbers of sequences used for phylogenetic trees.

| **PCR** | **Organism** | **Forward primer** | **Reverse Primer** | **Reference** |
| --- | --- | --- | --- | --- |
| SSU rRNA | Microsporidia | CACCAGGTTGATTCTGCC | TTATGATCCTGCTAATGGTTC | 1 |
| *rpoB* MB | *Microsporidia MB* | ACAGTAGGTCACTTGATTGAATGTC | TACCATGTGCTTAAGTCTTTGGT | This study |
| SINE S200 X6.1 | *Anopheles* | TCGCCTTAGACCTTGCGTTA | CGCTTCAAGAATTCGAGATAC | 2 |
| MB *18S* | *Microsporidia MB* | CGCCGGCCGTGAAAAATTTA | CCTTGGACGTGGGAGCTATC | This study |
| *Ribosomal Protein S7* | *Anopheles* | TCCTGGAGCTGGAGATGAAC | GACGGGTCTGTACCTTCTGG | 3 |
| *CytB* | *Plasmodium* | TGGTAGCACAAATCCTTTAGGG | TGGTAATTGACATCCAATCC | 4 |
|  |  |  |  |  |
|  |  |  |  |  |
| **FISH** | **Organism** | **Probe** |  | **Reference** |
| MB probe | *Microsporidia MB* | CCCTGTCCACTATACCTAATGAACAT |  | This study |

Supplementary Table 5: Primers used for PCR-based detection, quantification, gene expression analysis and Fluorescence in Situ Hybridization (FISH).

Supplementary references:

1. Ghosh, K. & Weiss, L. M. Molecular Diagnostic Tests for Microsporidia. *Interdiscip Perspect Infect Dis*. 2009, 926521 (2009).

2. Santolamazza, F., Mancini, E., Simard, F., Qi, Y., Tu, Z., della Torre, A. Insertion polymorphisms of SINE200 retrotransposons within speciation islands of Anopheles gambiae molecular forms. *Malar. J.* **7**, 163 (2008).

3. Dong, Y., Taylor, H.E., Dimopoulos, G. AgDscam, a hypervariable immunoglobulin domain-containing receptor of the *Anopheles gambiae* innate immune system. *PLoS Biol.* **4,** e229 (2006).

4. Xu, W. et al. SYBR green real-time PCR-RFLP assay targeting the Plasmodium cytochrome B gene - A highly sensitive molecular tool for malaria parasite detection and species determination. *PLoS ONE* **10**, e0120210 (2015).
