# Supplementary material for: A microsporidian impairs Plasmodium falciparum transmission in Anopheles arabiensis mosquitoes
Source: Nat Commun. 2020 May 4;11:2187. doi: 10.1038/s41467-020-16121-y (PMC7198529; doi:10.1038/s41467-020-16121-y)
Supplement: Supplementary file 3 — Reporting Summary [file 41467_2020_16121_MOESM3_ESM.pdf]

natureresearch

Corresponding author(s): Jeremy K. Herren

Last updated by author(s): March 11 2020

Reporting Summary

Nature Research wishes to improve the reproducibility of the work that we publish. This form provides structure for consistency and transparency in reporting. For further information on Nature Research policies, see Authors & Referees and the Editorial Policy Checklist .

Statistics

For all statistical analyses, confirm that the following items are present in the figure legend, table legend, main text, or Methods section.

n/a	Confirmed	
☐	☒	The exact sample size (n) for each experimental group/condition, given as a discrete number and unit of measurement
☐	☒	A statement on whether measurements were taken from distinct samples or whether the same sample was measured repeatedly
☐	☒	The statistical test(s) used AND whether they are one- or two-sided<br>*Only common tests should be described solely by name; describe more complex techniques in the Methods section.*
☒	☐	A description of all covariates tested
☒	☐	A description of any assumptions or corrections, such as tests of normality and adjustment for multiple comparisons
☐	☒	A full description of the statistical parameters including central tendency (e.g. means) or other basic estimates (e.g. regression coefficient) AND variation (e.g. standard deviation) or associated estimates of uncertainty (e.g. confidence intervals)
☐	☒	For null hypothesis testing, the test statistic (e.g. F, t, r) with confidence intervals, effect sizes, degrees of freedom and P value noted<br>*Give P values as exact values whenever suitable.*
☒	☐	For Bayesian analysis, information on the choice of priors and Markov chain Monte Carlo settings
☒	☐	For hierarchical and complex designs, identification of the appropriate level for tests and full reporting of outcomes
☒	☐	Estimates of effect sizes (e.g. Cohen's d, Pearson's r), indicating how they were calculated

Our web collection on statistics for biologists contains articles on many of the points above.

Software and code

Policy information about availability of computer code

Data collection	No software was used.
Data analysis	Data was analyzed in Geneious Prime 2020.3, R 3.5.3, Image J 1.50i, Graphpad Prizm 6.0c, Minimap 2, MUSCLE 3.5, Samtools 1.6, QGIS 3.6.2.

For manuscripts utilizing custom algorithms or software that are central to the research but not yet described in published literature, software must be made available to editors/reviewers. We strongly encourage code deposition in a community repository (e.g. GitHub). See the Nature Research guidelines for submitting code & software for further information.

Data

Policy information about availability of data

All manuscripts must include a data availability statement. This statement should provide the following information, where applicable:

- Accession codes, unique identifiers, or web links for publicly available datasets
- A list of figures that have associated raw data
- A description of any restrictions on data availability

All data is available in the main text and supplementary information. The source data used to generate Figures 2-6 and Supplementary information Figures 1-3 and Supplementary information Tables 1-3 is available in the Source Data file. The Microsporidia MB 18S partial gene sequence has been submitted to Genbank with accession number MT160806[https://www.ncbi.nlm.nih.gov/nuccore/MT160806]. The Anopheles arabiensis RNA sequencing data has been submitted to the NCBI Sequence Read Archive (SRA) database under accession number PRJNA622655 [https://www.ncbi.nlm.nih.gov/sra/?term=PRJNA622655]

Field-specific reporting

Please select the one below that is the best fit for your research. If you are not sure, read the appropriate sections before making your selection.

☒ Life sciences ☐ Behavioural & social sciences ☐ Ecological, evolutionary & environmental sciences

For a reference copy of the document with all sections, see nature.com/documents/nr-reporting-summary-flat.pdf

Life sciences study design

All studies must disclose on these points even when the disclosure is negative.

Sample size	Sample size was not pre-determined using statistical methods. Numbers of samples were determined based on maximum feasibility and previous experience with other symbiotic microbes that block disease transmission. Experiments were repeated to ensure differences between groups were observed in repeated experiments.
Data exclusions	We only excluded data from failed experiments, generally caused by high mortality in both treatment and control mosquitoes.
Replication	We have been able to successfully repeat all experiments at least 3 times. All replicates were true biological replicates.
Randomization	Samples were allocated into groups based on infection status. Where mixed pools were generated, mosquito lines were randomly combined.
Blinding	Specific blinding was not used because the data collected was not subjective. However, samples were allocated codes so it would not have been obvious to the researchers carrying out assays which samples were control or treatment.

Reporting for specific materials, systems and methods

We require information from authors about some types of materials, experimental systems and methods used in many studies. Here, indicate whether each material, system or method listed is relevant to your study. If you are not sure if a list item applies to your research, read the appropriate section before selecting a response.

Materials & experimental systems

n/a	Involved in the study	
☒	☐	Antibodies
☒	☐	Eukaryotic cell lines
☒	☐	Palaeontology
☐	☒	Animals and other organisms
☐	☒	Human research participants
☒	☐	Clinical data

Methods

n/a	Involved in the study	
☒	☐	ChIP-seq
☒	☐	Flow cytometry
☒	☐	MRI-based neuroimaging

Animals and other organisms

Policy information about studies involving animals; ARRIVE guidelines recommended for reporting animal research

Laboratory animals	laboratory animals were not used.
Wild animals	Wild mosquitoes were collected from field sites in Kenya. An. arabiensis were collected inside houses and sheds using CDC light traps and by manual aspiration. All mosquitoes were transported by road from the field to the icipe-Thomas Odhiambo Campus (iTOC) laboratories and insectaries alive in cages, each sample represents an individual wild caught mosquito.
Field-collected samples	Adult mosquitoes were reared at 30°C (+/- 2°C) and 70% humidity with a constant 12-hour day / night cycle. The adult mosquitoes were fed on 6% glucose soaked in cotton wool.
Ethics oversight	Scientific review for the study was provided by the icipe scientific review committee.

Note that full information on the approval of the study protocol must also be provided in the manuscript.

Human research participants

Policy information about studies involving human research participants

Population characteristics	A total 0f 1811 children participated in the study. The mean age in the study group was 7.9 years. The participants were 51% male and 49% female. 38 (2%) of the participants (22M and 16F) were gametocyte positive, and the mean gametocyte density was 47.4 per µl of blood.

Recruitment	Recruitment of participants was not subject to bias. Recruitment for membrane feeding assay was based on Plasmodium gametocyte presence.
Ethics oversight	Ethics review was provided by the Kenya Medical Research Institute Scientific and Ethics Review Unit: KEMRI/RES/7/3/1 and Glasgow MLVS College Ethics Committee: Project Number 200170001.

Note that full information on the approval of the study protocol must also be provided in the manuscript.
